# Hybrid Advanced Oxidation Processes Involving Ultrasound: An Overview

**DOI:** 10.3390/molecules24183341

**Published:** 2019-09-13

**Authors:** Jagannathan Madhavan, Jayaraman Theerthagiri, Dhandapani Balaji, Salla Sunitha, Myong Yong Choi, Muthupandian Ashokkumar

**Affiliations:** 1 Solar Energy Lab, Department of Chemistry, Thiruvalluvar University, Vellore 632115, Tamilnadu, India; baladgp@gmail.com; 2 Centre of Excellence for Energy Research, Sathyabama Institute of Science and Technology, Deemed to be University, Chennai 600119, India; j.theerthagiri@gmail.com; 3 Department of Chemistry and Research Institute of Natural Sciences, Gyeongsang National University, Jinju 52828, Korea; mychoi@gnu.ac.kr; 4 Department of Chemistry, Sathyabama Institute of Science and Technology, Deemed to be University, Chennai 600119, India; sunithasalla@gmail.com; 5 School of Chemistry, University of Melbourne, Parkville campus, Melbourne, VIC 3010, Australia

**Keywords:** advanced oxidation process, organic pollutants, sonolysis, sonophotocatalysis, ultrasonics

## Abstract

Sonochemical oxidation of organic pollutants in an aqueous environment is considered to be a green process. This mode of degradation of organic pollutants in an aqueous environment is considered to render reputable outcomes in terms of minimal chemical utilization and no need of extreme physical conditions. Indiscriminate discharge of toxic organic pollutants in an aqueous environment by anthropogenic activities has posed major health implications for both human and aquatic lives. Hence, numerous research endeavours are in progress to improve the efficiency of degradation and mineralization of organic contaminants. Being an extensively used advanced oxidation process, ultrasonic irradiation can be utilized for complete mineralization of persistent organic pollutants by coupling/integrating it with homogeneous and heterogeneous photocatalytic processes. In this regard, scientists have reported on sonophotocatalysis as an effective strategy towards the degradation of many toxic environmental pollutants. The combined effect of sonolysis and photocatalysis has been proved to enhance the production of high reactive-free radicals in aqueous medium which aid in the complete mineralization of organic pollutants. In this manuscript, we provide an overview on the ultrasound-based hybrid technologies for the degradation of organic pollutants in an aqueous environment.

## 1. Introduction

Environmental pollutioncaused distinctively by anthropogenic sources is a common concern for the human race over the past few decades that have led to the exhaustion of the natural resources, viz., air, water, land, and soil, etc. Rapid industrialization and mindless disposal of wastes by humans has begun to exhibit deleterious consequences on Mother Nature, thus posing environmental degradation as a threatening affair. Improper treatment and indiscriminate release of poisonous aromatic organic compounds like phenols, benzene, chloro-aromatic chemicals, etc., by many industries are mainly blamed for water pollution. Effluents released from these industries usually contain an assortment of obstinate organic molecules of varying toxicity along with few inorganic molecules and heavy metals, whose presence may contribute to cancer and mutagenic effects in humans and aquatic organisms [1,2,3,4,5,6,7,8]. Due to the unpredictable nature of these pollutants, it has always been hard to devise one kind of treatment option that totally aids in covering a wide range of organic contaminants. For instance, physical strategies like, adsorption on activated charcoal, reverse osmosis and flocculation are regarded as non-destructive methods, but these processes move the pollutants from one phase to other, thus offering to increase additional contamination by leaving all these organic pollutants remaining on the Earth. This could possibly exhibit negative effects on both human health as well as the environment. Therefore, designing of more eco-friendly techniques to eradicate these environmental pollutants has turned into a challenging assignment for researchers worldwide.

**Figure 1:**
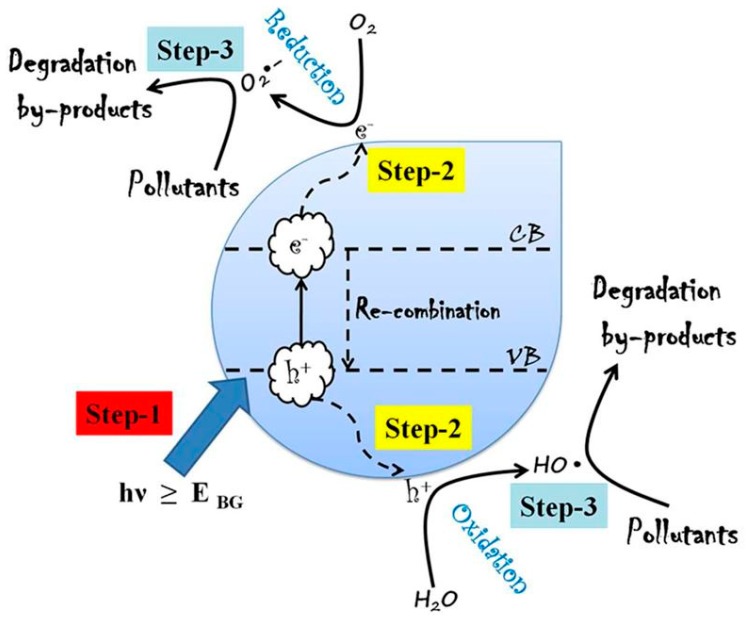
Schematic overview of photocatalytic degradation process [36]. Copyright (2019) Elsevier.

In this regard, advanced oxidation processes (AOPs) are recognised as an efficient method for the degradation of organic pollutants as they rely on the generation of highly reactive HO^•^ (hydroxyl radical) via chemical, solar or other types of energy options [9,10,11]. These radicals subsequently react with contaminants through a series of multi-step reactions to form low molecular weight carboxylic acids as end products and these products will further be mineralized into CO_2_ and H_2_O [12]. In general, AOPs involve ultraviolet (UV)-based processes (UV/O_3_/H_2_O_2_), chemical oxidation processes (O_3_/H_2_O_2_), Fenton and photo-Fenton processes (Fe^2+^/H_2_O_2_/UV), photocatalytic oxidation–reduction processes (semiconductor/UV or ultraviolet–visible (UV–vis)), critical water oxidation, sonolysis, and electron beams [13,14,15,16]. Of all these, photocatalysis is generally preferred for its ultimate mineralization and outstanding degradation capability of converting toxic organic compounds to CO_2_ and H_2_O. This process prompts a significant decrease of toxic heavy metal existence/conversion into non-toxic states, the destruction/deactivation of almost all water borne microorganisms, breaks down air pollutants, NO_2_, CO and NH_3_, and degrades plastics and a variety of industrially significant synthetic substances [17,18,19]. The photocatalysis process is reported to involve reduction and oxidation responses on the surface of photocatalyst material, mediated by the valence band (VB)holes(h^+^) and conduction band (CB) electrons (e^−^) produced by the absorption of UV–visible light. Such photo-created sets of h^+^ and e^-^ incites the development of powerful species, like, HO^•^ and superoxide radicals from atmospheric oxygen and moisture [20,21,22,23,24]. These species were reported to have the potentiality to oxidize and break down organic pollutants/poisonous gas/eliminate microorganisms from an aqueous environment. The most interesting component of AOPs is that they are profoundly intense in the generation of oxidising radicals and thus enabling the destruction of a wide range of obstinate organic pollutants with no selectivity. There are numerous reports on the generation of HO^•^ involving AOPs towards the degradation of organic pollutants [25,26,27,28,29,30,31,32,33,34,35]. Other than HO^•^, AOPs generate hydroperoxyl (HO_2_^•^) radicals which also aid the degradation of inorganic and organic compounds present in industrial wastewater. Figure 1 depicts a schematic overview of photocatalytic degradation process [36]. As depicted in Figure 1, (i) photon energy higher than the band gap excites the photocatalyst and creates e^-^ and h^+^, (ii) pollutants are adsorbed on the surface of photocatalyst and subsequently the oxygen molecules undergo reduction. At the same time, h^+^ reacts with adsorbed water molecules to produce HO^•^ that can oxidize harmful compounds [37,38]. The most widely studied semiconductor metal oxide photocatalysts are TiO_2_, ZnO, Fe_2_O_3_ and WO_3_ [39,40,41,42,43,44,45].

Likewise, the removal/destruction of the organic pollutants by means of acoustic cavitation, i.e., the use of ultrasound, is another efficient AOP [27,28,29,30,31,32,33,34,35,46,47,48,49]. The utilization of ultrasound in AOPs, for the degradation of organic pollutants, is found to be technically and economically more viable. The selectivity and enhanced reactivity are also main advantages of employing ultrasound in AOPs.

**Figure 2:**
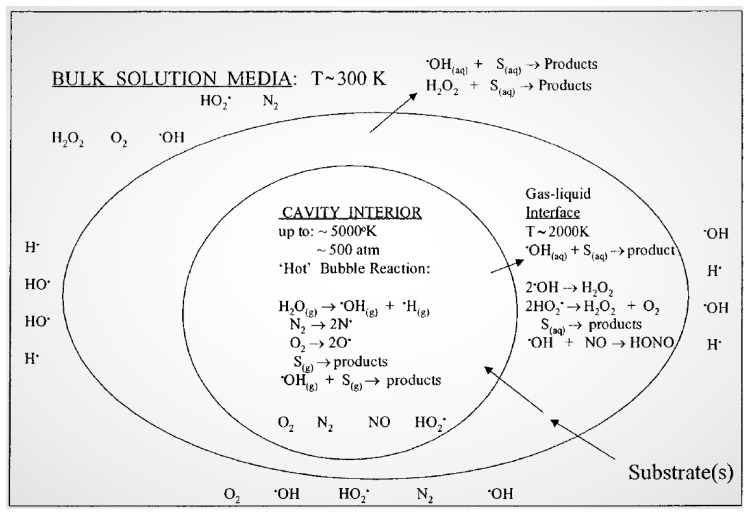
The sonochemical process of three different cavitaion zones [49]. Copyright (2019) American Chemical Society.

Generally, the sonochemical responses occur in three zones: centre of cavitation bubble (cavity interior), bubble–fluid interface (Gas-liquid interface), and bulk solution (Figure 2) [11,49].

1. Cavity interior (gaseous region): in this zone, hydrophobic and volatile molecules are degraded due to high temperature. The cavitation bubbles produce free radicals such as HO^•^ and H^•^ by the pyrolysis of water molecules.
2. Gas–liquid interface: the primary radicals produced inside the cavitation bubbles can react with solutes adsorbed at the cavitation bubble and solution interface, thus originating the degradation progress.
3. Bulk solution: the free radicals move from the gas–liquid interface into the bulk solution to produce secondary sonochemical reactions. Subsequently, the degradation reaction pathway occursbased on the pollutant nature such as solubility, volatility and surface action.

## 2. Ultrasound-Based Hybrid Advanced Oxidation Processes (AOPs)

Ultrasound (US) was first used by Richards and Loomis [37] for creating the concept of cavitation and degassing of water by accelerating the chemical reactions. US has since been used for many applications such as cell disruption, crystallization, atomization, degassing, polymerization, emulsification, nanotechnology, wastewater treatment, chemical reactions, food preservation, drug delivery and many more [50]. The major advantages of ultrasonic irradiation are safety, high penetrability in water medium, high degradation efficiency and the use of relatively low energy [51,52]. Being an extensively used AOP, many recent publications provide good reviews on the use of sonochemistry for wastewater treatment aspects [53,54,55,56]. Babu et al. [57] reported on combined AOPs such as sonolysis, sono-ozone process, sonophotocatalysis, sono-Fenton systems and sonophoto-Fenton methods for the degradation of pollutants. Other reports on combination of US with one or more AOPs were found to exhibit enhanced performance than that of individual AOPs. This may be due to the synergistic effects of the hybrid AOPs. The application of ultrasound is not only used in the preparation of active catalysts but has also been extensively applied in wastewater treatment applications [58].

## 3. Sonolysis

**Figure 3:**
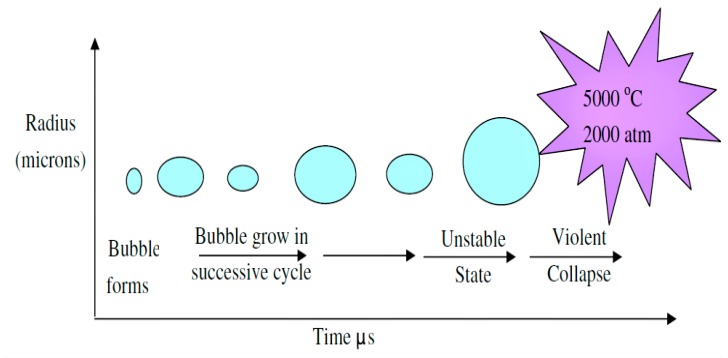
Ultrasound (US) waves induced cavitation occurrence and its collapse [61]. Copyright (2019) Elsevier.

Sonolysis is the process of utilization of ultrasonic irradiation without the presence of catalysts, to produce HO^•^ in aqueous media. It is one of the successful systems utilized for the degradation of organic pollutants in water [59,60]. The interaction between sound energy and dissolved bubbles in liquids leads to the growth of bubbles and subsequent near-adiabatic collapse, a process known as acoustic cavitation (Figure 3) [61]. Acoustic cavitation generates extreme temperature and pressure conditions within the collapsing bubbles [62,63,64]. In water, acoustic cavitation process generates HO^•^ radicals that could be used for the degradation of organic pollutants [65,66,67].

**Figure 4:**
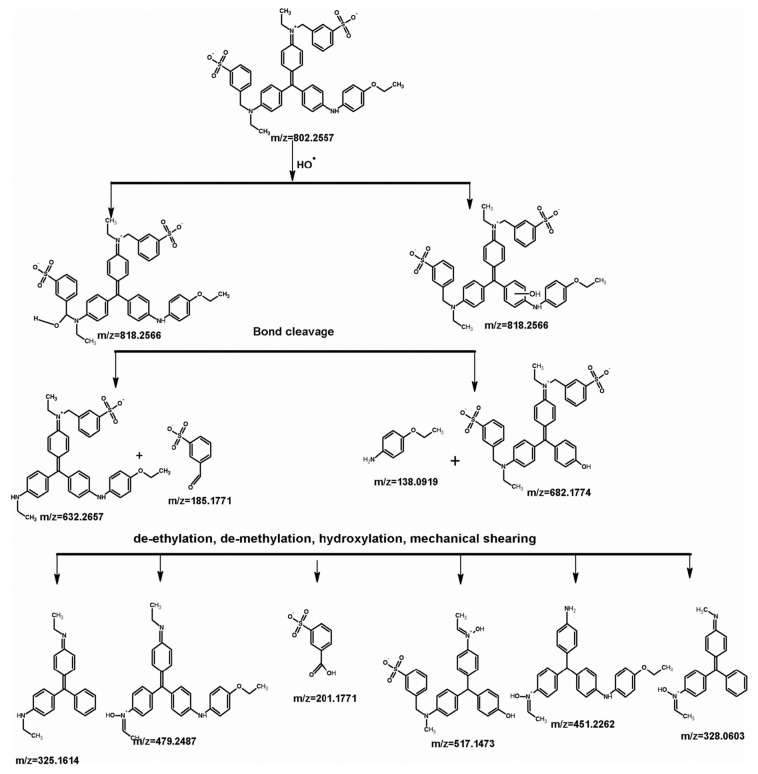
The degradation mechanistic reaction pathway of coomassie brilliant blue (CBB) [70]. Copyright (2019) Springer Nature.

Nair et al. [68] reported on the sonolysis treatment of dye-contaminated wastewater by two methods, i.e., probe-type sonicator (named as dipping process) and bath-type sonicator (named as without dipping process) maintained at an optimal pH of 6.0. The results of sonication treatment without dipping showed 71% chemical oxygen demand (COD) removalin 90 min, whereas, the sonication treatment with dipping showed a maximum COD removal of 82%. As a result of their investigation, they reported that sonolysis with a dipping process was demonstrated to be competent for degradation of organic pollutants. Pharmaceutical effluent containing β-blocker agent like atenolol was subjected to sonochemical degradation in an aqueous electrolyte as reported by Nejumal et al. [69]. In this study, four different ultrasound frequencies, 200, 350, 620 and 1 MHz, were employed to degrade atenolol. A maximum degradation of 90% was noted at a frequency of 350 kHz. The respective sonolysis degradation was studied in terms of total organic carbon (TOC), COD reduction and ion chromatography (IC) and high-performance liquid chromatography (HPLC) analysis. The cavitation efficiency was found to be maximum at 200 kHz, which could be due to a combination of radical yield per bubble and the total number of active bubbles. Rayaroth et al. [70] reported on the degradation of coomassie brilliant blue (CBB) by an ultrasonic irradiation technique. Nearly 90% of degradation was noted in pure water after 30 min at 350 kHz frequency and 60 W power. Degradation studies conducted in sea water also showed similar results after 90 min of sonication. The comparative results of pure and sea water showed a time dependent COD removal as 94 and 48%, respectively. The degradation mechanistic reaction pathway of CBB is shown in Figure 4.

At different concentration and operational conditions, the naphthol blue black (NBB) reduction rate was monitored by Dalhatou et al. [71] using sonochemical degradation at 278 kHz. The inorganic ions like bicarbonate and phosphate ions were found to play a role in maintaining a stable pH which in turn influenced the interfacial radical species generation and increased the rate of degradation. Rahmani et al. [72] reported a sonolysis treatment for tinidazole degradation by the combination of ultrasound and H_2_O_2_. They reported that the production of H_2_O_2_ combined with ultrasonic irradiation process exhibited better degradation results due to the formation of higher amount of hydroxyl radicals. Drijvers et al. [73] proposed the sonolysis treatment of the four monohalo-generated benzene products such as fluoro-benzene (FB), chloro-benzene (CB), bromo-benzene (BB) and iodo-benzene (IB) at different initial concentrations under optimized conditions. A range of ultrasonic frequencies were extensively used to report on the effective degradation of organic compounds [74,75,76,77,78,79,80,81,82,83,84,85,86,87,88,89].

However, ultrasonic process is a high-energy process. When the volume of contaminated water is large, the process may not be energy efficient.

## 4. Sonocatalysis

**Figure 5:**
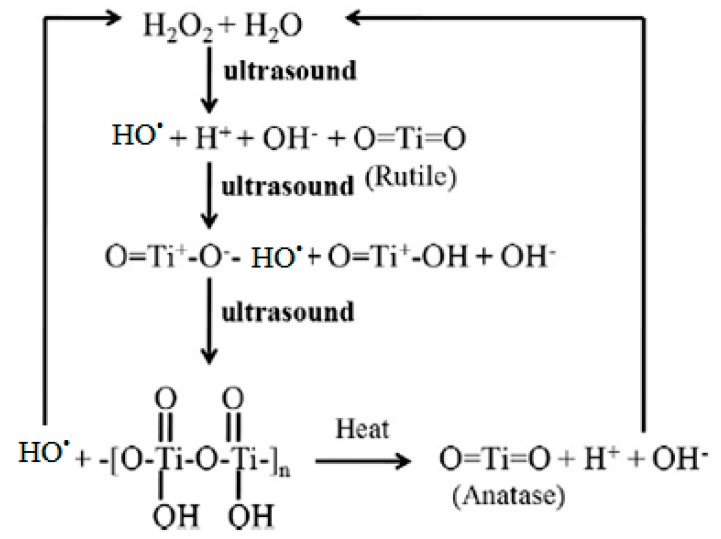
The probable conversion of rutile to anatase TiO_2_ under ultrasonic irradiation [102]. Copyright (2019) Elsevier.

Rapid growth in nanotechnology has gained a great deal of interest in environmental applications. Nanomaterials in various shapes/morphologies/forms have a significant impact on the treatment of water and air quality in natural environment [90,91,92]. A combination of nanocatalysts and ultrasonication to create heterogeneous sonocatalytic processes advances the degradation efficiency of organic pollutants to a huge extent. The sonocatalysts offer improved mass transfer owing to a larger surface area. Furthermore, they also increase the number of cavitation bubbles by acting as nucleation sites. The presence of catalysts can increase the quantity of free radicals generated, thus enhancing the rate of degradation of organic pollutants [93,94,95,96,97,98,99,100,101,102,103,104,105]. An acoustic cavitation process is also useful to generate active catalysts. For example, the conversion of rutile to anatase TiO_2_ under ultrasonic irradiation is shown in Figure 5 [102].

Khataee et al. [106] prepared ZrO_2_ on pumice and tuff by using a modified sol-gel process and utilized for sonocatalytic degradation of rifampin (RIF). Zaman et al. [107] used highly active SnO_2_/MWCNT nanocomposite for the sonocatalytic degradation of methylene blue (MB). A higher degradation efficiency was obtained with SnO_2_/MWCNT nanocomposite (98%) compared to that of pure SnO_2_ (48%) and MWCNT (64%). The enhanced degradation performance of composite was due to a synergistic effect by the coupling of SnO_2_ and MWCNT, which promoted the transfer of electrons from SnO_2_ to MWCNT. Khataee et al. [108] synthesized TiO_2_-biochar (TiO_2_-BC) composite using a sol-gel method and utilized for the sonocatalytic degradation of reactive blue 69 (RB69) dye. The optimized parameters for an efficient degradation were found to be neutral pH, catalyst amount of 1.5 g/L, initial concentration of dye 20 mg/L and ultrasonic intensity of 300 W.The higher sonocataytic degradation of the nanocomposite was due to the formation of sonochemical hot spots. Khataee et al. [109] developed Gd_x_Zn_1-x_O composite for the degradation of acid orange 7 (AO7) using sonocatalytic process at pH 7. The optimized 5% Gd-doped ZnO composite showed a higher sonocatalytic performance of about 90% at 90 min. It was noted that the degradation percentage was decreased from 90% to 56% after the addition of sodium carbonate, sodium sulfate and sodium chloride due to the interfering reactions by the additives.

Siadatnasab et al. [110] synthesized CuS/CoFe_2_O_4_ (CuS/CFO) hybrids using hydrothermal method and utilized as a sonocatalyst for the degradation of methylene blue. The degradation efficiency under sonolysis/H_2_O_2_ was compared with different sonocatalysts, viz., CuS/H_2_O_2_, CFO/H_2_O_2_ and CuS/CFO/H_2_O_2_. These systems showed 6%, 62%, 23% and 100% degradation efficiency, respectively within a reaction time of 30 min for MB. The synergistic integration of H_2_O_2_ and sonocatalyst dosage was found to favour more hydroxyl radical formation that resulted in higher degradation of MB. Lee et al. [111] designed a GO/β-Bi_2_O_3_/TiO_2_/Bi_2_Ti_2_O_7_ (GBT) nanocomposite via a two-step hydrothermal reduction process for the sonocatalytic degradation of pharmaceuticals such as carbamazepine (CBZ) and acetaminophen (ACE). The degradation performance was carried out at various operating frequenciessuch as 28, 580, and 970 kHz at power density level of 180 W/L and also compared with pristine catalysts such as Bi-doped GO and Ti-doped GO. It was noticed that the highest photocatalytic degradation performance was observed at an optimized frequency at 580 kHz and GBT catalysts. Furthermore, the degradation of CBZ was found to be higher than that of ACE due to its high hydrophobicity.

## 5. Sonophotocatalysis

**Figure 6:**
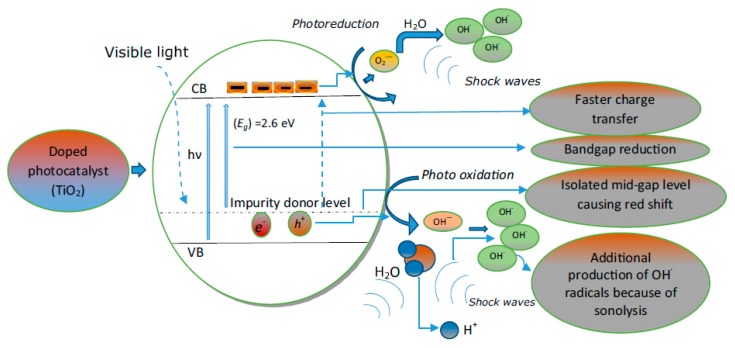
Graphical representation of synergetic effect during a sonophotocatalytic degradation process using doped semiconductor oxide [58]. Copyright (2019) Elsevier.

Sonolysis has received much attention towards the organic pollutant degradation due to its simplicity. The photocatalytic process is considered as one of the greatest technologies for wastewater treatment processes. While sonolysis is an efficient process for the degradation of hydrophobic pollutants, its efficiency towards hydrophilic compounds is weak [58]. Hydrophobic compounds adsorb to the cavitation bubble interface and are efficiently attached by HO^•^ radicals generated on bubble collapse. On the other hand, photocatalysis is an efficient process for the degradation of hydrophilic compounds since they have preferential adsorption to the relatively polar catalytic surfaces. Hence, a combination of sonolysis and photocatalysis, sonophotocatalysis, would help to overcome the disadvantages of the individual processes and synergistically combine the advantages of these processes. In addition, an acoustic cavitation process would clean the surface of photocatalysts regenerating the active sites and the surface of catalysts would act as cavitation bubble nucleation sites. As can be seen from the above discussion, sonophotocatalysis is expected to be more efficient due to a number of synergistic effects [58]. A schematic overview representation of synergetic effect during sonophotocatalysis using doped semiconductor metal oxide is shown in Figure 6 [58].

Madhavan et al. [3] reported the degradation of paracetamol by sonophotocatalysis using TiO_2_ as a photocatalyst, which exhibited a higher degradation performance than that of individual processes. The rates of degradation observed for photocatalysis, sonolysis and sonophotocatalysiswere about 30.2, 8.3 and 40.2 × 10^−7^ M/min, respectively. The observed enhancement was due to the formation of additional hydroxyl radicals via the excitation of the visible light active iron-aqua complex in water. Madhavan et al. [4] studied the degradation of acid red 88 (AR88) by photocatalysis, sonolysis and sonophotocatalysis processes using TiO_2_ photocatalysis. The degradation process of AR88 with combined techniques of UV + TiO_2_, US + TiO_2_ and US+UV+TiO_2_ was carried out with an initial concentration of 0.09 mM AR88 and 1 g/L of photocatalyst. The results showed the sonophotocatalysis with TiO_2_ exhibited an enhanced degradation activity due to positive synergetic effect with synergy index of 1.3, demonstrating the coupling of sonolysis and photocatalysis as a potential process for AR88 degradation. Madhavan et al. [5] also investigated the degradation of metanate hydrochloride (FMT) using various processes such as, sonolytic, photocatalytic and sonophotocatalytic processes with Fe^3+^ and TiO_2_ catalysts using ultrasound of 213 kHz. The rates of degradation attained for photocatalysis, sonolysis and sonophotocatalysis with 1 g/L of TiO_2_ were about 32.6, 5.1 and 28.1 × 10^−7^ M/min, respectively. The coupling of TiO_2_ photocatalysis and sonolysis exhibited a negative synergy effect with the index of 0.7. However, the rates of degradation attained using UV+Fe^3+^, US+Fe^3+^ and US+UV+Fe^3+^ were 10.1, 14.1 and 40.9 × 10^−7^ M/min, respectively. The sonophotocatalysis process using Fe^3+^ exhibited a positive synergy with index of 1.6. The degradation of diclofenac using photocatalysis, sonolysis and sonophotocatalysis was studied [14] using ZnO as a photocatalyst. The degradation efficiency of the observed process of photocatalysis, sonolysis, sonophotocatalysis were about 68%, 23% and 73%, respectively. The enhancement was attributed to the continuous cleaning of the photocatalyst surface to produce more hydroxyl radicals. The mineralization and degradation of orange-G using sonophotocatalysis with TiO_2_ as a photocatalyst was also investigated [15]. The results inferred that the solution pH played an important role on the degradation. The sonophotocatalytic degradation of orange-G was comparatively higher at pH of 5.8 than 12, which indicate that an acidic medium was more favoured for the degradation of orange-G with a TOC reduction of 82% at pH 5.8. At neutral pH, the pollutant showed a relatively higher surface activity.

Vinoth et al. [112] reported on an assembled p-type NiO on n-type TiO_2_ to synthesiseTiO_2_-NiO nanocomposites with p-n junction via a US-mediated wet impregnation protocol. The sonophotocatalytic efficiencies of the pure TiO_2_ and the as-synthesisednano composites were studied under diffused sunlight employing methyl orange (MO) as an organic pollutant. The impregnation of NiO on to TiO_2_ was found to enhance the optical absorption at 500–800 nm (visible region) due to p-n junction formation at the interface. The degradation efficiency of MO by individual processes of photocatalysis and sonolysis under diffused sunlight was found to be 6% and 30%, respectively. A 4.8-fold increase was noted on combining the above processes due to their synergistic effect. The sonophotocatalytic activity of TiO_2_-NiO photocatalysts at different NiO loading was also studied and found that 10 wt% NiO loading to be the optimal range.

**Figure 7:**
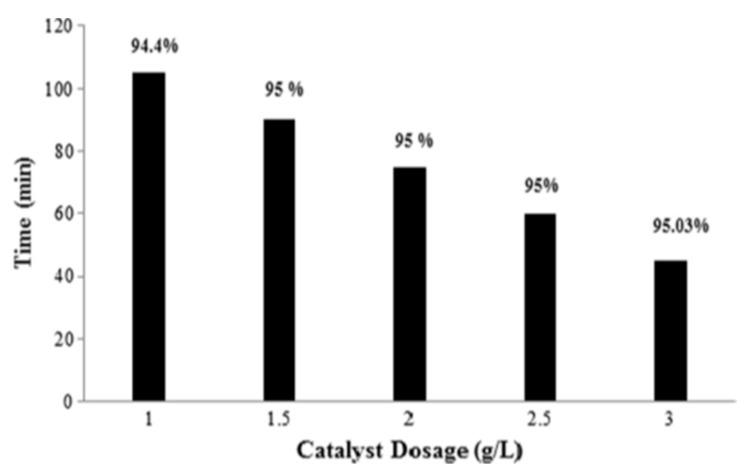
Effect of catalyst dosage on percentage colour removal of MB dye [116]. Copyright (2019) Elsevier.

Paul et al. [113] prepared an Ag-doped h-MoO_3_ sonophotocatalyst using a solvent-based self-assembly technique and the catalyst was tested for MB degradation in the presence of diffused sunlight. An improved degradation efficiency of MB was noticed when the doped Ag was added onto MoO_3_ surface. In addition to HO^•^ radicals, mechanical agitation and mass transfer effects were also reported to increase the sonophotocatalytic degradation of MB.Gokul et al. [114] developed a reduced graphene oxide supported binary metal oxide (TiO_2_-CuO/rGO) nanoparticle as a multilayer thin film for enhanced sonophotocatalytic degradation of MO using UV irradiation. The degradation level of MO was investigated by UV–visible and TOC measurements and 62% mineralisation was achieved. The increased rate of degradation was due to efficient electron transport by multi-layered TiO_2_/CuO/rGO. Sonophotocatalytic remediation of synthetic dye and textile effluent using Fe-doped Bi_2_O_3_ was reported by Dinesh et al. [115]. The effect of optimized experimental parameters, viz., initial pH, gas bubbling (oxygen, argon, air, and nitrogen) and oxidant addition (H_2_O_2_ and peroxymonosulfate) on the degradation efficacy of 10 ppm of Basic Brown 1 dye showed a maximum decolourization of 62% with 3 g/L peroxymono sulfate at 37 kHz frequency. A higher dye degradation efficiency of 99% was noted for sonophotocatalys using Fe-doped Bi_2_O_3_ catalyst with peroxymonosulfate. The higher degradation rate was reported due to the formation of both HO^•^ and SO_4_^•−^ radical species. Balakumara et al. [116] investigated the sonophototocatalyticdecolourization of MB in the presence of ZnO/Bi_2_O_3_ synthesised using a hydrothermal method. An enhanced decolourization was observed in a sonophotocatalytic process performed under optimized experimental parameters. The effect of ZnO/Bi_2_O_3_ catalyst dosage on decolourization percentage of MB dye is shown in Figure 7. It was observed that the decolourization increased as the amount of catalyst was increased, owing to the increased surface area of the catalyst and reduced recombination rate by the successful formation of heterojunction between two semiconductors of ZnO to Bi_2_O_3_.

**Figure 8:**
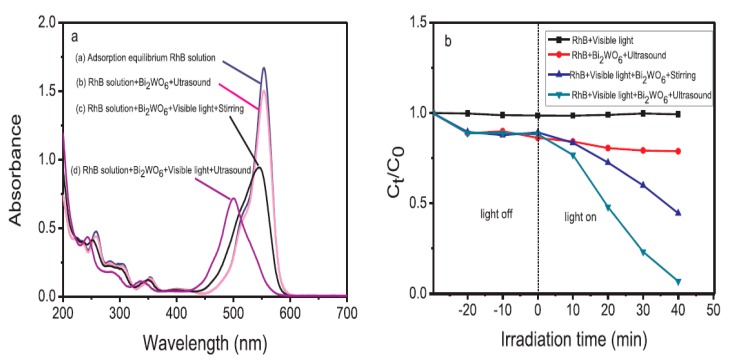
(**a**) Ultraviolet–visible (UV–vis) spectra of RhB with Bi_2_WO_6_ at various conditions (**b**) Degradation of RhB by different degradation processes at 40 min [120]. Copyright (2019) Elsevier.

Dhanasekar et al. [117] reported a high MB degradation using β-NiMoO_4_ sonocatalyst synthesized by a simple hydrothermal process in the presence of diffused sunlight. Hu et al. [118] prepared a stable and low toxicity sonophotocatalyst made of polyethylene glycol-modified TiO_2_ porous thin film for wastewater treatment. They reported 95% degradation of rhodamine B (RhB) using optimized PEG_2000_-TiO_2_ film under UV irradiation for 60 min. The combined photocatalytic-ultrasonic system with a high amount of PEG_2000_-TiO_2_ coated glass beads was found to exhibit a stronger ultrasonic efficiency. A longer irradiation time resulted in a higher RhB degradation efficiency due to the synergetic effect of the combined sonolysis and photocatalysis. Kumar et al. [119] investigated a Bi-doped TiO_2_ catalyst prepared by a sol-gel method and tested its efficiency on MB degradation in aqueous medium by hydrodynamic cavitation (HC) integrated with H_2_O_2_. About 95% degradation efficiency was reported after 60 min, which can be attributed to the synergetic effect of the photocatalytic process. Liang et al. [120] fabricated high-performance Bi_2_WO_6_ sonophotocatalyst in the presence of polyvinylpyrrolidone prepared by an optimized hydrothermal method and tested against the degradation of RhB with or without visible light under various experimental conditions. An excellent sonophotocatalytic activity of 100% degradation at 40 min was noted for RhB degradation, which was enhanced by an improved active surface site of Bi_2_WO_6_ during the sonophotocatalysis process. The related plots are shown in Figure 8.

Sunasee et al. [121] assessed a commercially available titanium dioxide (TiO_2_, P25) for the degradation of bisphenol A (BPA) using energy-based advanced oxidation process by combining US and UV. The degradation kinetic rate was also observed at optimal experimental parameters viz, power (50 W), frequency (35 kHz), temperature (20 °C) and mechanical stirring (300 rpm). The combined approach of US/UV/P25 was reported to exhibit higher sonophotocatalytic degradation efficiency with 96% of BPA (28.0 × 10^−3^ min^−1^) than sonolysis and photocatalysisover2 h. The by-products and the intermediates of the BPA were also analysed using HPLC–mass spectrometry (MS). The observed enhancement was due to the synergistic effect of the combined process.

## 6. Fenton, Sono-Fenton, and Sonophoto-Fenton Processes

Zhang et al. reported on the decolourization of C.I acid orange 7, by Fenton process in combination with ultrasound/goethite/H_2_O_2_ [122]. The decolourization was found to be affected by ultrasonic power density, goethite addition, and hydrogen peroxide concentration. The decolorization took place on the goethite surface and achieved a high performance with 90% of decolorization efficiency over ultrasound/goethite/H_2_O_2_, which is higher than that of the Fenton process without ultrasound (42%) with an optimal pH of 3 of the dye. Mishra et al. [123] investigated p-nitrophenoldegradation by sonophotocatalytic process with hydrogen peroxide and Fenton chemistry was carried out under the combination of US irradiation (25 kHz) with 1 kW of acoustic power and UV radiation (11 W). A maximum p-nitrophenol degradation efficiency of 94% was noted with combined sonophotocatalysis and optimal concentration of H_2_O_2_. Fenton chemistry was reported to play a role in improving the extent of degradation. Taghizade et al. [124] discussed the sonophotocatalytic degradation of chitosan by sono-Fenton and sonophoto-Fenton processes in the presence of Fe (III)/H_2_O_2_ as a catalyst. The operating conditions of ultrasound irradiation were 24 kHz and 16W. The combined system of Fe (III) (2.5 × 10^−4^ mol/L) and H_2_O_2_ (0.020–0.118 mol/L) operated under UV irradiation showed a degradation rate of 1.873 × 10^−9^–6.083 × 10^−9^ mol/L/s. However, the photo-Fenton process achieved complete degradation of chitosan in 60 min with an increased rate and sufficient catalyst concentration. The efficiency of the photo-Fenton system achieved was related to formation more hydroxyl radicals through the active participation of iron in the redox cycle.

Zhou et al. [125] described the degradation kinetics of sodium alginate (NaAlg) via photo-Fenton, sono-Fenton, and sonophoto-Fenton processes in the presence of TiO_2_ nanoparticles. The experimental results obtained suggested that the photocatalytic degradation seemed to increase with increasing TiO_2_ concentration from 0.05 to 0.5 gL^−1^. The sonophoto-Fenton process showed a better NaAlg degradation result, which was due to the more positive synergy between ultrasound and TiO_2_photocatalysis. Zhi et al. [126] synthesized a RG-I enriched ultra-low molecular weight pectin by an ultrasound-accelerated Fenton process. An enhanced degradation efficiency of pectin (5.5 kD) was achieved in 35 min by Fenton reaction in the presence of US. In addition, the antioxidant activity of the US-Fenton-treated pectin was found to be significantly elevated. The combined process of ultrasound and Fenton (US-Fenton process) enhanced the degradation efficiency and achieved 5.2 kDa products in 1 h, which was higher than the Fenton (0.5 mM Fe^2+^ and 6 g/L H_2_O_2_) process which degraded from 448 kDa to 19.78 kDa,. The enhanced performance was due to the positive synergistic effect of the combined process, which produced more active free radicals.

**Figure 9:**
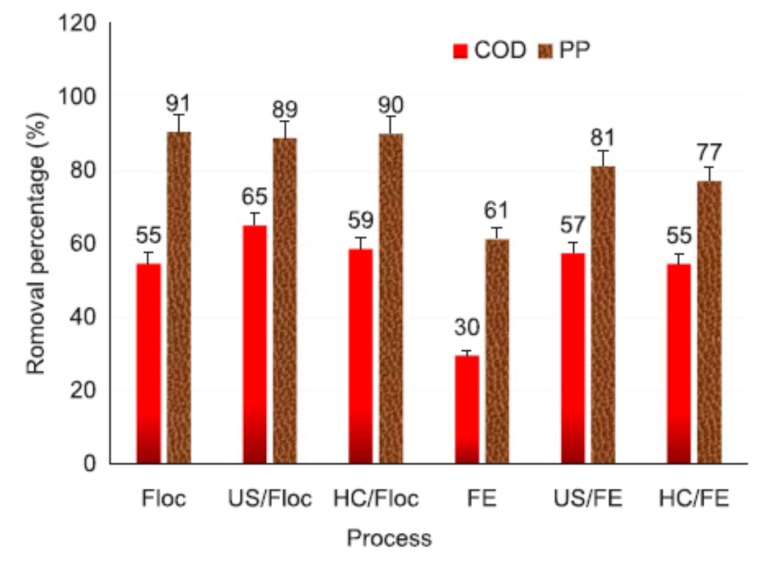
Comparison of chemical oxygen demand (COD) and polyphenol (PP) removal after Fenton- and flocculation-based pre-treatments [127]. Copyright (2019) Elsevier.

Wu et al. [127] investigated the effect of US and hydrodynamic cavitation (HC) to treat cork wastewater (CW) by flocculation (Floc) and Fenton processes. The pre-treatment by Fenton oxidation (FE) of the diluted CW showed COD and polyphenol (PP) removal efficiency of 30% and 61%, respectively, while optimized HC and US showed an 83%–90% increase in COD reduction and a 26%–33% increase in polyphenol reduction. While the flocculation process was found to show 55% and 91% of COD and polyphenol removal. The physical and chemical effects of the cavitational collapse was found to responsible for the efficient removal of PP. The plot of COD and PP removal efficiency using Floc and FE processes with and without US and HC is shown Figure 9. It can be seen that the FE process alone provided comparatively low COD and PP removal of 30% and 61%, respectively. However, when coupled with US and HC, it showed enhanced COD and PP removal (Figure 9). The enhanced removal might be due to the cavitation effects causing the production of more reactive species, such as H_2_O_2_ and HO^•^ radicals.

Petrier et al. [104] reported on phenol degradation by the hydroxyl radicals at an acoustic power of 50 W and at a frequency range of 20 to 500 kHz. An enhanced degradation rate was noted on the addition of Fenton’s reagent at a lower frequency of 35 kHz. By contrast, Namkung et al. [105] observed no appreciable enhancement in TOC removal during phenol degradation using advanced Fenton by altering the sonication intensity from the power level 2.4 to 4.7 W in acidic medium containing ferrous and zero valent iron as a Fenton’s type reagent. However, on increasing the H_2_O_2_ flow rate from 14 to 60 mL/h, a four-fold improvement in the TOC removal from 11% to 38% was observed, which evidenced the role of H_2_O_2_ as the most important factor responsible for enhanced degradation. ElMetwally et al. [128] reported the role of metal oxychlorides(FeOCl (I), CuOCl (II), ZnOCl (III) and BiOCl (IV)) in heterogeneous Fenton-sonophotocatalytic degradation of nitrobenzene at room temperature and pH 7.0 in the presence of US (20 kHz), UV (6W, λ = 254 nm) and combined UV/US irradiation in the presence of H_2_O_2_. The degradation was achieved with a higher mineralization percentage for the combined process of US and UV, than that of pure UV and US processes. The order of degradation was found to be US/UV > UV > US with the mineralization extents of 46%, 41%, 35% and 33%, respectively, under the combined irradiation of US/UV for 60 min. The improved degradation rate was due to the production of more reactive free radicals and positive synergistic effect.

In addition to the above studies, the stability and reusability of a catalyst is significant for the commercial and industrial applications. The catalyst can be recovered after the completion of each cycle (sonophotocatalysis, sonocatalysis, and photocatalysis processes), dried, and reused.

## 7. Summary

A brief overview of the various methods involving sonolysis for the effective degradation of organic contaminants in homogenous and heterogeneous solutions has been provided with specific examples. The ultrasonic process for removal of organic contaminants is an efficient method because of its non-toxic and non-selective features. However, utilization of the ultrasonic process as an individual technique has some disadvantages for large-scale applications due to the low efficiency for the removal of certain organic contaminants, mainly, hydrophilic compounds. The application of ultrasound in combination with photocatalysis, photolysis, and Fenton processes may offer synergistic effects. In photocatalysis, the disadvantages such as inactiveness towards hydrophobic pollutants and catalyst poisoning due to product adsorption, etc. could be overcome by the combined process of sonophotocatalysis. The technique of sonophotocatalysis can be an efficient process for the removal of toxic pollutants. However, an extensive investigation is required for the utilization of a wide-range of visible light-active catalysts in the sonophotocatalytic process for effective large-scale applications. While numerous reports are available in the literature on lab-scale studies, the use of combined AOPs in industrial processes has not yet been investigated.

## References

[1] Díaz-Cruz M.S., Barceló D. (2008). Trace organic chemicals contamination in ground water recharge. Chemosphere.

[2] Mohapatra D.P., Brar S.K., Tyagi R.D., Surampalli R.Y. (2010). Physico-chemical pretreatment and biotransformation of wastewater and wastewater sludge-fate of bisphenol A. Chemosphere.

[3] Madhavan J., Grieser F., Ashokkumar M. (2013). Sonophotocatalytic degradation of paracetamol using TiO_2_ and Fe^3+^. Sep. Purif. Technol..

[4] Madhavan J., Kumar P.S., Anandan S., Grieser F., Ashokkumar M. (2010). Degradation of acid red 88 by the combination of sonolysis and photocatalysis. Sep. Purif. Technol..

[5] Madhavan J., Grieser F., Ashokkumar M. (2010). Sonophotocatalytic degradation of Formetanate hydrochloride using homogeneous and heterogenousphotocatalysts. Sep. Purif. Technol..

[6] Madhavan J., Kumar P.S., Grieser F., Ashokkumar M., Anandan S. (2010). Sonophotocatalytic degradation of diclofenac using doped and undoped semiconductor nanopartilces. Chemosphere.

[7] Madhavan J., Kumar P.S., Grieser F., Ashokkumar M., Anandan S. (2010). Sonophotocatalytic degradation of monocrotophos using TiO_2_ and Fe^3+^. J. Hazard. Mater..

[8] Belvar C., Bellod R., Fuerte A., Garcia M.F. (2006). Nitrogen-containing TiO_2_ photocatalysts: Part Synthesis and solid characterization. Appl. Catal. B Environ..

[9] Kudo T., Nakamura Y., Ruike A. (2003). Development of rectangular column structured titanium oxide photocatalysts anchored on silica sheets by a wet process. Res. Chem. Intermed..

[10] Bahnemann D. (2004). Photocatalytic water treatment: Solar Energy Applications. Solar Energy.

[11] Theerthagiri J., Senthil R.A., Thirumalai D., Madhavan J. (2016). Sonophotocatalytic Degradation of Organic Pollutants Using Nanomaterials. Handbook of Ultrasonics and Sonochemistry.

[12] Carp O., Huisman C.L., Reller A. (2004). Photoinduced reactivity of titanium dioxide. Prog. Solid State Chem..

[13] Choi H., Al-Abed S.R., Dionysiou D.D., Stathatos E., Lianos P. (2010). TiO_2_-Based Advanced Oxidation Nanotechnologies for Water Purification and Reuse: Sustainability Science and Engineering.

[14] Khataee A.R., Kasiri M.B. (2010). Artificial neural networks modeling of contaminated water treatment processes by homogeneous and heterogeneous nanocatalysis. J. Mol. Catal. A Chem..

[15] Madhavan J., Greiser F., Ashokkumar M. (2010). Degradation of Orange G by advanced oxidation processes. Ultrason. Sonochem..

[16] Madhavan J., Maruthamuthu P., Ashokkumar M., Murugesan S. (2009). Kinetics of degradation of acid red 88 in presence of Co^2+^-ion/peroxomonosulfate reagent. Appl. Catal. A Gen..

[17] Theerthagiri J., Senthil R.A., Priya A., Madhavan J., Muthupandian A. (2015). Synthesis of visible-light active V_2_O_5_/g-C_3_N_4_ composite photocatalyst. New J. Chem..

[18] Theerthagiri J., Senthil R.A., Madhavan J., Neppolian B. (2015). A comparative study on the role of precursors of graphitic carbon nitrides for the photocatalytic degradation of direct red 81. Mater. Sci. Forum..

[19] Sherine O.O., Gerald J.M. (2004). Nanostructured Materials for Environmental Remediation of Organic Contaminants in Water. J. Environ. Sci. Health Part A Toxic/Hazard. Subst. Environ. Eng..

[20] Theerthagiri J., Senthil R.A., Priya A., Madhavan J., Michael R.J.V., Ashokkumar M. (2014). Photocatalytic and photoelectrochemical studies of Visible-light active α-Fe_2_O_3_-g-C_3_N_4_nanocomposites. RSC Adv..

[21] Theerthagiri J., Senthil R.A., Malathi A., Selvi A., Madhavan J., Ashokkumar M. (2015). Synthesis and characterization of CuS-WO_3_ composite photocatayst for enhanced visible light photocatalytic activity. RSC Adv..

[22] Khin M.M., Nair A.S., Babu V.J., Rajendran M., Ramakrishna S. (2012). A review on nanomaterials for environmental remediation. Energy Environ. Sci..

[23] Theerthagiri J., Senthil R.A., Senthilkumar B., Polu A.R., Madhavan J., Ashokkumar M. (2017). Recent advances in MoS_2_ nanostructured materials for energy and environmental applications—A Review. J. Solid State Chem..

[24] Micheal K., Ayeshamariam A., Boddula R., Arunachalam P., Al-Salhi M.S., Theerthagiri J., Prasad S., Madhavan J., Al-Mayouf A.M., Mariam A. (2019). Assembled composite of hematite iron oxide on sponge-like BiOCl with enhanced photocatalytic activity. Mater. Sci. Energy Technol..

[25] Khataee A.R., Pons M.N., Zahraa O. (2009). Photocatalytic degradation of three azo dyes using immobilized TiO_2_ nanoparticles on glass plates activated by UV light irradiation: Influence of dye molecular structure. J. Hazard. Mater..

[26] Khataee A., Vatanpour V., Ghadim A.A., Khataee A. (2009). Decolorization of C.I. Acid Blue 9 solution by UV/nano-TiO_2_, Fenton, Fenton-like, electro-Fenton and electrocoagulation processes: A comparative study. J. Hazard. Mater..

[27] Flint E.B., Suslick K.S. (1991). The temperature of cavitation. Science.

[28] Mason T.J., Lorimer J.P. (1988). Sonochemistry (Theory, Applicationsand Uses of Ultrasound in Chemistry).

[29] Adewuyi Y.G., Appaw C. (2002). Sonochemical oxidation of carbon di sulphide in aqueous solutions: Reaction kinetics and pathways. Ind. Eng. Chem. Res..

[30] Appaw C., Adewuyi Y.G. (2002). Destruction of carbon disulfide in aqueous solutions by sonochemical oxidation. J. Hazard. Mater. B.

[31] Ley S.V., Low C.M.R. (1989). Ultrasound in Synthesis, Reactivity and Structure: Concepts in Organic Chemistry.

[32] Lu Y., Weavers L.K. (2002). Sonochemical desorption and destruction of 4-chlorobiphenyl from synthetic sediments. Environ. Sci. Technol..

[33] Hua I., Hoffmann M.R. (1997). Optimization of Utrasonic radiation as an advanced oxidation technology. Environ. Sci. Technol..

[34] Gogate P.R., Pandit A.B. (2004). Sonophotocatalytic Reactors forWastewater Treatment: A Critical Review. AIChE J..

[35] Madhavan J., Greiser F., Ashokkumar M. (2009). Degradation of Orange G by sonophoto Fenton process. Water Sci. Technol..

[36] Malathi A., Arunachalam P., Madhavan J., Ashokkumar M. (2018). A review on BiVO_4_ photocatalyst: Morphology control, activity enhancement for solar photocatalytic applications. Appl. Catal. A Gen..

[37] Richards W.T., Loomis A.L. (1927). The chemical effects of high frequency sound waves I: A preliminary survey. J. Am. Chem. Soc..

[38] Deng Y., Zhao R. (2015). Advanced Oxidation Processes (AOPs) in Wastewater Treatment. Curr. Pollut. Rep..

[39] Kansal S.K., Singh M., Sud D. (2007). Studies on photodegradation of two commercial dyes in aqueous phase using different photocatalysts. J. Hazard. Mater..

[40] Gouvêa C.A., Wypych F., Moraes S.G., Durán N., Nagata N., Peralta-Zamora P. (2000). Semiconductor-assisted photocatalytic degradation of reactive dyes in aqueous solution. Chemosphere.

[41] Neppolian B., Choi H.C., Sakthivel S., Banumathi A., Murugesan V. (2002). Solar/UV-induced photocatalytic degradation of three commercial textile dyes. J. Hazard. Mater..

[42] Cristian L., Juanita F., Jaime B., Mansilla H.D. (2002). Optimized photodegradation of Reaction Blue 19 on TiO_2_ and ZnO. Catal. Today.

[43] Rajat A., Jitendra V., Punjabi P.B., Ameta S.C. (2006). Use of semiconducting iron(III) oxide in photocatalytic bleaching of some dyes. Ind. J. Chem. Technol..

[44] Li W., Allioux F.M., Lee J., Ashokkumar M., Dumée L.F. (2018). Ultrasound-assisted fabrication of metal nano-porous shells across polymer beads and their catalytic activity for reduction of 4-nitrophenol. Ultrason. Sonochem..

[45] Theerthagiri J., Sunitha S., Senthil R.A., Nithyadharseni P., Madankumar A., Arunachalam P., Maiyalagan T., Kim H.S. (2019). A review on ZnO nanostructured materials: Energy, environmental and biological applications. Nanotechnology.

[46] Colmenares J.C., Xu Y.J. (2016). Heterogeneous Photocatalysis: From Fundamentals to Green Applications.

[47] Chatel G., Colmenares J.C. (2017). Sonochemistry: From Basic Principles to Innovative Applications.

[48] Chatel G., Behling S.V.R., Colmenares J.C. (2017). A Combined Approach using Sonochemistry and Photocatalysis: How to Apply Sonophotocatalysis for Biomass Conversion?. ChemCatChem.

[49] Adewuyi Y.G. (2001). Sonochemistry: Environmental Science and Engineering Applications. Ind. Eng. Chem. Res..

[50] Wang J., Jiang Y., Zhang Z., Zhao G., Zhang G., Ma T., Sun W. (2007). Investigation on the sonocatalytic degradation of congo red catalyzed by nanometer rutile TiO_2_ powder and various influencing factors. Desalination.

[51] Bruce D.A., Nareddy A., Lee S. (2006). Sonochemical Reaction Engineering, Encyclopedia of Chemical Processing.

[52] Hoffmann M.R., Hua I., Höchemer R. (1996). Application of ultrasonic irradiation for the degradation of chemical contaminants in water. Ultrason. Sonochem..

[53] Adewuyi Y.G. (2005). Sonochemistry in environmental remediation I: Combinative and hybrid sonophotochemical oxidation processes for the treatment of pollutants in water. Environ. Sci. Technol..

[54] Adewuyi Y.G. (2005). Sonochemistry in environmental remediation II: Heterogeneous sonophotocatalytic oxidation processes for the treatment of pollutants in water. Environ. Sci. Technol..

[55] Gonzalez-Garcia J., Saez V., Tudela I., Diez-Garcia M.I., Esclapez M.D., Louisnard O. (2010). Sonochemical treatment of water polluted by chlorinated organo compounds. Water.

[56] Chowdhury P., Viraraghavan T. (2009). Sonochemical degradation of chlorinated organic compounds, phenolic compounds and organic dyes: A review. Sci. Total Environ..

[57] Babu S.G., Ashokkumar M., Neppolian B. (2016). The role of ultrasound on advanced oxidation processes. Top. Curr. Chem..

[58] Panda D., Manickam S. (2017). Recent advancements in the sonophotocatalysis (SPC) and doped-sonophotocatalysis (DSPC) for the treatment of recalcitrant hazardous organic water pollutants. Ultrason. Sonochem..

[59] Ollis D.F., Al-Ekabi H. Photocatalytic purification and treatment of water and air. Proceedings of the 1st International Conference on TiO_2_ Photocatalytic Purification and Treatment of Water and Air.

[60] Asim N., Badeiei M., Ghoreishi B.K., Ludin N.A., Reza M. (2012). New developments in photocatalysts modification: Case study of WO_3_. Advances in Fluid Mechanics and Heat & Mass Transfer, Proceedings of the 10th WSEAS International Conference on Heat Transfer, Thermal Engineering and Environment (HTE ‘12), Istanbul, Turkey, 21–23 August 2012.

[61] Pilli S., Bhunia P., Yan S., LeBlanc R.J., Tyagi R.D., Surampalli R.Y. (2011). Ultrasonic retreatment of sludge: A review. Ultrason. Sonochem..

[62] Thangavadivel K., Konagay M., Okitsu K., Ashokkumar M. (2014). Ultrasound-assisted degradation of methyl orange in a micro reactor. J. Environ. Chem. Eng..

[63] Margulis M.A., Margulis I.M. (2004). Mechanism of sonochemical reactions and sonoluminescence. High Energy Chem..

[64] Entezari M.H., Heshmati A., Yazdi S. (2005). A combination of ultrasound and inorganic catalyst: Removal of 2-chlorophenol from aqueous solution. Ultrason. Sonochem..

[65] Henglein A., Gutierrez M. (1988). Sonolysis of polymers in aqueous solution. New observations on pyrolysis and mechanical degradation. J. Phys. Chem..

[66] Mason T.J., Lorimer J.P., Bates D.M., Zhao Y. (1994). Dosimetry in sonochemistry: The use of aqueous terephthalate ion as a fluorescence monitor. Ultrason. Sonochem..

[67] Mason T.J., Bernal V.S., Pollet B.G. (2012). An introduction to sonoelectrochemistry. Power Ultrasound in Electrochemistry.

[68] Nair R.R., Patel R.L. (2014). Treatment of Dye Wastewater by Sonolysis Process. IJRMEET.

[69] Nejumal K.K., Manoj P.R., Aravind U.K., Aravindakumar C.T. (2014). Sonochemical degradation of a pharmaceutical waste, atenolol, in aqueous medium. Environ. Sci. Pollut. Res..

[70] Rayaroth M.P., Aravind U.K., Aravindakumar C.T. (2017). Ultrasound based AOP for emerging pollutants: From degradation to mechanism. Environ. Sci. Pollut. Res..

[71] Dalhatou S., Pétrier C., Laminsi S., Baup S. (2015). Sonochemical removal of naphthol blue black azo dye: Influence of parameters and effect of mineral ions. Int. J. Environ. Sci. Technol..

[72] Rahmani H., Gholami M., Mahvi A.H., Alimohammadi M., Azarian G., Esrafili A., Rahmani K., Farzadkia M. (2014). Tinidazole Removal from Aqueous Solution by Sonolysisin the Presence of Hydrogen Peroxide. Bull. Environ. Contam. Toxicol..

[73] Drijvers D., Langenhove H.V., Herrygers V. (2000). Sonolysis of fluoro-, chloro-, bromo-and iodobenzene: A comparative study. Ultrason. Sonochem..

[74] Kidak R., Ince N.H. (2006). Ultrasonic destruction of phenol and substituted phenols: A review of current research. Ultrason. Sonochem..

[75] Liu H., Liang M.Y., Liu C.S., Gao Y.X., Zhou J.M. (2009). Catalytic degradation of phenol in sonolysis by coal ash and H_2_O_2_/O_3_. Chem. Eng. J..

[76] Ku Y., Tu Y.H., Ma C.M. (2005). Effect of hydrogen peroxide on the decomposition of monochlorophenols by sonolysis in aqueous solution. Water Res..

[77] Lim M., Son Y., Yang J., Khim J. (2008). Addition of chlorinated compounds in the sonochemical degradation of 2-chlorophenol. Jpn. J. Appl. Phys..

[78] Francony A., Petrier C. (1996). Sonochemical degradation of carbon tetrachloride in aqueous solution at two frequencies: 20 kHz and 500 kHz. Ultrason. Sonochem..

[79] Wang J., Sun W., Zhang Z., Zhang X., Li R., Ma T., Zhang P., Li Y. (2007). Sonocatalytic degradation of methyl parathion in the presence of micron-sized and nano-sized rutile titanium dioxide catalysts and comparison of their sonocatalytic abilities. J. Mol. Catal. A Chem..

[80] Li J., Cai J., Fan L. (2008). Effect of sonolysis on kinetics and physicochemical properties of treated chitosan. J. Appl. Polym. Sci..

[81] Bahena C.L., Martinez S.S., Guzman D.M., Hernandez M.D. (2008). Sonophotocatalytic degradation of alazine and gesaprim commercial herbicides in TiO_2_ slurry. Chemosphere.

[82] Nanzai B., Okitsu K., Takenaka N., Bandow H. (2009). Sonochemical degradation of alkylbenzenesulfonates and kinetics analysis with a langmuir type mechanism. J. Phys. Chem. C.

[83] David B. (2009). Sonochemical degradation of PAH in aqueous solution. Part I: Monocomponent PAH solution. Ultrason. Sonochem..

[84] Zhang H., Jiang M., Wang Z., Wu F. (2007). Decolorisation of CI Reactive Black 8 by zerovalent iron powder with/without ultrasonic irradiation. Color. Technol..

[85] Wang J., Wang X., Guo P., Yu J. (2011). Degradation of reactive brilliant red K-2BP in aqueous solution using swirling jet-induced cavitation combined with H_2_O_2_. Ultrason. Sonochem..

[86] Priya M.H., Madras G. (2006). Kinetics of TiO_2_-catalyzed ultrasonic degradation of Rhodamine dyes. Ind. Eng. Chem. Res..

[87] Torres-Palma R.A., Serna-Galvis E.A. (2018). Chapter 7 Sonolysis. Advanced Oxidation Processes for Waste Water Treatment.

[88] Vadivel S., Theerthagiri J., Madhavan J., Maruthamani D. (2016). Synthesis of polyaniline/graphene oxide composite via ultrasonication method for photocatalytic applications. Mater. Focus.

[89] Sivalingam G., Nagaveni K., Hegde M.S., Giridhar M. (2003). Photocatalytic degradation of various dyes by combustion synthesized nanoanatase TiO_2_. Appl. Catal. B Environ..

[90] Li J., Xu Y., Liu Y., Wu D., Sun Y. (2004). Synthesis of hydrophilic ZnSnanocrystals and their application in photocatalytic degradation of dye pollutants. China Particuol..

[91] Sajjadi S., Khataee A., Kamali M. (2017). Sonocatalytic degradation of methylene blue by a novel graphene quantum dots anchored CdSenanocatalyst. Ultrason. Sonochem..

[92] Mehrizad A., Behnajady M.A., Gharbani P., Sabbagh S. (2019). Sonocatalytic degradation of Acid Red 1 by sonochemically synthesized zinc sulfide-titanium dioxide nanotubes: Optimization, kinetics and thermodynamics studies. J. Clean. Prod..

[93] Pang Y.L., Abdullah A.Z. (2012). Comparative study on the process behaviour and reaction kinetics in sonocatalytic degradation of organic dyes by powder and nanotubes TiO_2_. Ultrason. Sonochem..

[94] Wang J., Guo B., Zhang X., Zhang Z., Han J., Wu J. (2005). Sonocatalytic degradation of methyl orange in the presence of TiO_2_ catalysts and catalytic activity com-parison of rutile and anatase. Ultrason. Sonochem..

[95] Areerob Y., Cho J.Y., Jang W.K., Oh W.C. (2018). Enhanced sonocatalytic degradation of organic dyes from aqueous solutions by novel synthesis of mesoporous Fe_3_O_4_-graphene/ZnO@SiO_2_nanocomposites. Ultrason. Sonochem..

[96] Huang Y., Zhang H., Wei C., Li G., Wu Q. (2017). Assisted sonocatalytic degradation of pethidine hydrochloride (dolantin) with some inorganic oxidants caused by CdS coated ZrO_2_ composite. Sep. Purif. Technol..

[97] Meng Z.D., Zhu L., Choi J.G., Park C.Y., Oh W.C. (2012). Sonocatalytic degradation of Rhodamine B in the presence of C60 and CdS coupled TiO_2_ particles. Ultrason. Sonochem..

[98] Zhang H., Wei C., Huang Y., Wang J. (2016). Preparation of cube micrometer potassium niobate (KNbO_3_) by hydrothermal method and sonocatalytic degradation of organic dye. Ultrason. Sonochem..

[99] Thompson L.H., Doraiswamy L.K. (1999). Sonochemistry: Science and engineering. Ind. Eng. Chem. Res..

[100] Wang J., Jiang Y., Zhang Z., Zhang X., Ma T., Zhang G., Zhao G., Zhang P., Li Y. (2007). Investigation on the sonocatalytic degradation of acid red B in the presence of nanometer TiO_2_ catalysts and comparison of catalytic activities of anatase and rutile TiO_2_ powders. Ultrason. Sonochem..

[101] Wang J., Ma T., Zhang Z., Zhang X., Jiang Y., Dong D., Zhang P., Li Y. (2006). Investigation on the sonocatalytic degradation of parathion in the presence of nanometer rutile titanium dioxide (TiO_2_) catalyst. J. Hazard. Mater..

[102] Wang J., Ma T., Zhang Z., Zhang X., Jiang Y., Zhang G., Zhao G., Zhao H., Zhang P. (2007). Investigation on transition crystal of ordinary rutile TiO_2_ powder and its sonocatalytic activity. Ultrason. Sonochem..

[103] Wang J., Ma T., Zhang Z., Zhang X., Jiang Y., Pan Z. (2007). Preparation of high active nanometer TiO_2_sonocatalyst by partial transition crystal in hydrogen peroxide solution under ultrasonic irradiation. Catal. Commun..

[104] Petrier C., Francony A. (1997). Ultrasonic waste-water treatment: Incidence of ultrasonic frequency on rate of phenol and carbon tetrachloride degradation. Ultrason. Sonochem..

[105] Namkunga K., Burgess A., Bremner D., Staines H. (2007). Advanced Fenton processing of aqueous phenol solutions: A continuous system study including sonication effects. Ultrason. Sonochem..

[106] Khataee A., Gholami P., Kayan B., Kalderis D., Dinpazhoh L., Akay S. (2018). Synthesis of ZrO_2_ nanoparticles on pumice and tuff for sonocatalytic degradation of rifampin. Ultrason. Sonochem..

[107] Zaman S., Zhang K., Karim A., Xin J., Sun T., Gong J.R. (2017). Sonocatalytic degradation of organic pollutant by SnO_2_/MWCNT nanocomposite. Diam. Relat. Mater..

[108] Khataee A., Kayan B., Gholami P., Kalderis D., Akay S. (2017). Sonocatalytic degradation of an anthraquinone dye using TiO_2_-biochar nanocomposite. Ultrason. Sonochem..

[109] Khataee A., Soltani R.D.C., Karimi A., Joo S.W. (2015). Sonocatalytic degradation of a textile dye over Gd-doped ZnO nanoparticles synthesized through sonochemical process. Ultrason. Sonochem..

[110] Siadatnasab F., Farhadi S., Khataee A. (2018). Sonocatalytic performance of magnetically separable CuS/CoFe_2_O_4_nanohybrid for efficient degradation of organic dyes. Ultrason. Sonochem..

[111] Lee G., Chu K.H., Al-Hamadani Y.A., Park C.M., Jang M., Heo J., Her N., Kim D.H., Yoon Y. (2018). Fabrication of graphene-oxide/β-Bi_2_O_3_/TiO_2_/Bi_2_Ti_2_O_7_ heterojunctednanocomposite and its sonocatalytic degradation for selected pharmaceuticals. Chemosphere.

[112] Vinoth R., Karthik P., Devan K., Neppolian B., Ashokkumar M. (2017). TiO_2_–NiO p–n nanocomposite with enhanced sonophotocatalytic activity under diffused sunlight. Ultrason. Sonochem..

[113] Paul M., Dhanasekar M., Bhat S.V. (2016). Silver doped h-MoO_3_ nanorods for sonophotocatalytic degradation of organic pollutants in ambient sunlight. Appl. Surf. Sci..

[114] Gokul P., Vinoth R., Neppolian B., Anandhakumar S. (2016). Binary metal oxide Nanoparticle Incorporated Composite Multilayer Thin Films for Sono-Photocatalytic Degradation of Organic Pollutants. Appl. Surf. Sci..

[115] Dinesh G.K., Anandan S., Sivasankar T. (2016). Synthesis of Fe-doped Bi_2_O_3_ nanocatalyst and its sonophotocatalytic activity on synthetic dye and real textile wastewater. Environ. Sci. Pollut. Res. Int..

[116] Balakumara R., Sathya K., Saravanathamizhan R. (2016). Decolorization of Methylene Blue Dye Using Sonocatalytic Followed by Photocatalytic Process. Water Conserv. Sci. Eng..

[117] Dhanasekar M., Ratha S., Rout C.S., Bhat S.V. (2017). Efficient sono-photocatalytic degradation of methylene blue using nickel molybdatenanosheets under diffused sunlight. J. Environ. Chem. Eng..

[118] Hu X., Zhu Q., Gu Z., Zhang N., Liu N., Stanislaus M.S., Li D., Yang Y. (2016). Wastewater Treatment by Sonophotocatalysis using PEG modified TiO_2_ film in a circular photocatalytic-ultrasonic system. Ultrason. Sonochem..

[119] Kumar M.S., Sonawane S.H., Pandit A.B. (2017). Degradation of methylene blue dye in aqueous solution using hydrodynamic cavitation based hybrid advanced oxidation processes. Chem. Eng. Process..

[120] Liang L., Tursun Y., Nulahong A., Dilinuer T., Tunishaguli A., Gao G., Abulikemu A., Okitsu K. (2017). Preparation and sonophotocatalytic performance of hierarchical Bi_2_WO_6_ structures and effects of various factors on the rate of Rhodamine B degradation. Ultrason. Sonochem..

[121] Sunasee S., Wong K.T., Lee G., Pichiah S., Ibrahim S., Park C., Kim N.C., Yoo Y., Jang M. (2016). Titanium dioxide-based sonophotocatalytic mineralization of biphenyl A and its intermediates. Environ. Sci. Pollut. Res..

[122] Zhang H., Fu H., Zhang D. (2009). Degradation of C.I. Acid Orange 7 by ultrasound enhanced heterogeneous Fenton-like process. J. Hazard. Mater..

[123] Mishra K.P., Gogate P.R. (2010). Intensification of sonophotocatalytic degradation of p-nitrophenol at pilot scale capacity. Ultrason. Sonochem..

[124] Taghizade M.T., Abdollahi R., Orang N.S. (2012). Sonophotocatalytic degradation of Chitosan in the Presence of Fe (III)/H_2_O_2_ System. J. Polym. Environ..

[125] Zhou Q., Liu Y., Yu G., He F., Chen K., Xiao D., Zhao X., Feng Y., Li J. (2016). Degradation kinetics of sodium alginate via sono-Fenton, photo-Fenton and sono-photo-Fenton methods in the presence of TiO_2_ nanoparticles. Polym. Degrad. Stab..

[126] Zhi Z., Chen J., Li S., Wang W., Huang R., Liu D., Ding T., Linhardt R.J., Chen S., Ye X. (2017). Fast preparation of RG-I enriched ultra-low molecular weight pectin by an ultrasound accelerated Fenton process. Sci. Rep..

[127] Wu Z., Francisco J., Córdoba Y., Cintasd P., Wu Z., Boffa L., Mantegna S., Cravotto G. (2017). Effects of ultrasonic and hydrodynamic cavitation on the treatment of cork wastewater by flocculation and Fenton processes. Ultrason. Sonochem..

[128] ElMetwally A.E., Eshaq G.H., Al-Sabagh A.M., Yehia F.Z., Philip C.A., Moussa N.A., ElShafei G.M. (2018). Insight into heterogeneous Fenton-sonophotocatalytic degradation of nitrobenzene using metal oxychlorides. Sep. Purif. Technol..

